# Primary Care Perceptions Among Spanish-Speaking Populations: A Comprehensive Review

**DOI:** 10.7759/cureus.68736

**Published:** 2024-09-05

**Authors:** Angela D Quiroz, Raegan Boothe, Hannah Cruz, Sainamitha R Palnati, Saajan Bhakta

**Affiliations:** 1 Department of Behavioral Sciences/Department of Counseling, South Mountain Community College, Phoenix, USA; 2 Department of Research, Kansas College of Osteopathic Medicine, Wichita, USA

**Keywords:** access to care, cultural competence, healthcare outcomes, health disparities, interpreter services, language barriers, patient-provider communication, patient satisfaction, rural healthcare, spanish-speaking populations

## Abstract

This literature review explores the substantial impact of language barriers on healthcare outcomes for Spanish-speaking populations, emphasizing the need for improved language support systems. While this review emphasizes the growing Hispanic/Latino population in Kansas as a case study, the findings underscore broader challenges faced by individuals with limited English proficiency in accessing and utilizing healthcare services across similar rural settings in the United States. Language barriers hinder effective communication between patients and healthcare providers, affecting patient care, satisfaction, and outcomes. Despite federal regulations requiring language assistance, the availability and quality of interpreter services remain inconsistent, exacerbating healthcare disparities.

A comprehensive literature search was conducted across electronic databases including PubMed, SageJournals, Science Direct, and Springer Link for studies published from 2004 to 2024. The search was conducted from April 10, 2024 to May 31, 2024 using the following terms: "language concordance," "health outcomes," "Spanish, language barriers," "primary care," and "rural settings." The search terms were combined using Boolean operators: "Spanish OR Hispanic" for ethnic identification, "language concordance AND health outcomes" to explore the relationship between language alignment and patient results, and "Spanish AND primary care AND language barriers" to narrow the focus to specific healthcare settings.

The review calls for continued research and the implementation of robust language support systems to ensure equitable healthcare access and improved health outcomes for Spanish-speaking populations in rural Kansas.

## Introduction and background

Language barriers in healthcare present a significant challenge to the delivery of equitable and effective medical services, particularly among non-English-speaking populations. With an estimated 45 million immigrants, the United States of America (USA) is one of the most diverse countries in the world. Among the 66 million people in the USA who speak a language apart from English, approximately 38% have limited English proficiency (LEP) [1]. These statistics alone indicate the critical need for effective communication between healthcare workers and their patients. Language concordance, which refers to a situation where a healthcare provider and patient speak the same primary language, is essential in overcoming these barriers and ensuring effective communication [2]. Navigating the healthcare system can be a complex task for anyone but is especially challenging for individuals with LEP. These patients must be clearly informed about their diagnoses, treatment options, insurance coverage, and how to access healthcare services. Moreover, they should feel empowered to advocate for their health. However, language barriers often exacerbate difficulties, making medical care less accessible and potentially compromising the quality of care.

This review explores the importance of language concordance, the effectiveness of interpreter services, and the need for standardized assessment of clinician language proficiency. Additionally, it highlights the broader implications of cultural responsiveness in healthcare, emphasizing the need for healthcare systems to adapt to the linguistic and cultural diversity of their patient populations.

Language barriers impacting healthcare

Language differences between healthcare providers and patients significantly influence various aspects of patient care, including provider-patient relationships, healthcare outcomes, patient satisfaction, and healthcare utilization. Numerous studies have explored the specific ways in which language differences can positively or negatively affect these areas [3-10]. For instance, patients treated by physicians who spoke the same language as they did were less likely to miss medical appointments, spent less time in waiting rooms, and had fewer difficulties speaking with healthcare providers over the phone [11].

The impact of language differences on physicians has also been examined. Physicians who are proficient in Spanish, for example, are more likely to elicit comprehensive information and reach more accurate diagnoses in Spanish-speaking populations, even when compared to English-speaking physicians who use professional interpreting services [12]. Both Spanish-speaking patients and their healthcare providers reported higher levels of satisfaction when professional interpreter services were provided. Moreover, Spanish-speaking patients treated by physicians who spoke Spanish reported having greater autonomy in their medical care and treatment decision-making [3]. However, achieving language concordance is often challenging, especially in rural areas where bilingual clinicians are scarce.

Language barriers as a healthcare disparity

With a significant number of individuals in the USA having LEP, addressing language barriers is crucial in tackling healthcare disparities [1]. Effective communication is a cornerstone of quality healthcare, and interruptions caused by language barriers can quickly lead to adverse outcomes [2,6,7,11,13]. To receive medical care in the USA, many individuals rely on health insurance and referral systems. For informed consent to be meaningful, patients must understand their diagnoses and treatments. Language barriers can affect these aspects of care and disrupt the patient-physician relationship [4,12,14-16]. This relationship is essential for building trust and facilitating effective communication, both of which are critical to positive health outcomes [2,4,5,14]. Even when an interpreter is available, interactions may feel as though they are directed at the interpreter rather than between the patient and physician [16]. Additionally, misinterpretations can lead to delayed or inappropriate care [6,16]. Patients might also feel uncomfortable discussing sensitive health information in the presence of an interpreter, despite confidentiality assurances [2]. This discomfort is particularly evident when ad hoc interpreters are used, as patients may feel that the interpreter lacks the professional discretion expected in medical settings. For example, one patient reported feeling uneasy when a non-professional interpreter, who was also a staff member at the clinic, was brought in to assist, leading to a sense of lost trust and a breakdown in the direct patient-provider connection. The patient expressed that this added a barrier to discussing more personal and confidential matters, which would typically only be shared with the doctor [7].

Language barriers create significant obstacles at every stage of the healthcare process, starting even before a patient enters a facility. Obtaining health insurance or scheduling appointments can be daunting without language support, leading to delays in care. Once at the healthcare facility, patients may be unaware of or uncomfortable requesting interpreter services, as such information is often not prominently displayed or communicated [7]. This often forces reliance on limited English skills or untrained family members for translation.

In the examination room, communicating symptoms effectively can be challenging if language barriers are present. Misunderstandings regarding medical history and symptoms can lead to misdiagnosis or inappropriate treatment [7,13]. Even if diagnostic and treatment plans are explained, a lack of comprehension can leave patients uncertain about subsequent steps in their care [3]. At discharge, patients typically receive paperwork only in English, often without being informed that translated versions are available. This oversight can result in patients missing essential instructions and follow-up details, potentially leading to complications or inadequate care after discharge [13]. Picking up prescribed medications also poses challenges, as labels, instructions, and warnings are typically in English, increasing the risk of medication errors and adverse reactions [2]. Studies have shown that LEP patients are at a higher risk for medication errors due to misinterpretation of dosage instructions and misunderstandings of medication purposes. For example, in one study, a family who used an ad hoc interpreter mistakenly administered two tablespoons instead of the prescribed two teaspoons of medication to their child, which highlights the potential dangers when language barriers exist during medication administration​ [6].

These language barriers highlight the necessity for enhanced language support and interpreter services throughout the healthcare process to ensure equitable access and understanding for all patients. Non-English-speaking individuals in the USA still have healthcare needs; therefore, services must be provided in their native languages to achieve optimal health outcomes. The complexities of the healthcare system mean that language barriers can exacerbate disparities in access and outcomes.

Language concordance: Kansas as a case study

Kansas, with its rapidly growing Hispanic/Latino population, serves as a compelling case study for examining the impact of language barriers in rural healthcare settings. According to the United States Census Bureau, the Hispanic/Latino population in Kansas has increased by 25% from 2010 to 2020, representing 13% of the total state population [7]. This population growth is due in part to the availability of jobs at hog processing plants [17]. The challenges faced by Spanish-speaking populations in Kansas, particularly those with LEP, are reflective of broader trends observed in similar rural areas across the United States. By focusing on Kansas, this review not only sheds light on the specific difficulties encountered in this region but also provides insights that can inform strategies for addressing language barriers in other rural communities with comparable demographic shifts.

Research has explored the impacts of language barriers in healthcare among Kansas residents [7,8]. Many issues were highlighted by Spanish-speaking individuals with LEP, such as limited knowledge of how to pay for medical bills, inadequacy of interpreters, and medical errors they have experienced due to language barriers. Several federal regulations have been implemented to require healthcare institutions, specifically hospitals that accept Medicare or doctors who accept Medicaid, to provide qualified interpreters to patients with LEP. These requirements must be fulfilled according to the National Standards on Culturally and Linguistically Appropriate Services (CLAS) based on Title VI of the Civil Rights Act of 1964 and the non-discrimination provision to Section 1557 of the Affordable Care Act [7].

Title VI states that “no person in the United States shall, on the ground of race, color, or national origin, be excluded from participation in, be denied the benefits of, or be subjected to discrimination under any program or activity receiving federal financial assistance” [18].

The standards set by CLAS aim to ensure equitable access to services by mandating the availability of free language assistance and informing individuals of these services in their preferred language, both verbally and in writing. CLAS also emphasizes the importance of ensuring the competency of those providing language assistance and requires that print, multimedia materials, and signage be available in the languages commonly spoken by the populations in service areas [19].

In 2016, the non-discrimination provision (Section 1557) of the Affordable Care Act was updated to specify that interpreters must be ‘qualified’ rather than merely ‘competent,’ though it still does not mandate a standardized certification. Section 1557 also restricts the use of ad hoc interpreters to qualified individuals whose job responsibilities include interpretation. The use of minors, especially those related to the patient, such as children, as interpreters is prohibited, as is the use of adult family and friends, except in specific circumstances [7].

Two national certification programs for interpreters have also been established: the National Board of Certification for Medical Interpreters (NBCMI) and the Certification Commission for Interpreters in Health Care (CCIH). Despite these initiatives, individuals with LEP in Kansas continue to face challenges due to language barriers. In fact, no real standard has been defined for a person to become an interpreter. In Kansas, healthcare interpreters are not required to be certified or to have a minimum number of training hours, and no clear standard exists for qualifying as an interpreter [7]. Currently, as of 2024, only 45 individuals are registered as Certified Healthcare Interpreters (CCHI) in Kansas, with an additional 12 certified through the National Board of Certification for Medical Interpreters (NBCMI), across all languages [20,21]. Moreover, there is no guarantee that existing federal legislation regarding language services is consistently enforced in Kansas, despite efforts to monitor compliance [7]. Many Spanish-speaking patients with LEP in Kansas report feelings of disempowerment, discomfort, and misunderstanding during medical appointments [22]. These gaps in certification and enforcement exacerbate the challenges faced by Spanish-speaking patients with LEP, underscoring the need for improved language services.

Several key terms used in this publication that are important to properly define are listed in Table 1.

**Table 1:** Key terms and definitions. References: [2,3,5,13,22-26]

Key Terms	Definition
Limited English proficiency (LEP)	Individuals who have a limited ability to communicate effectively in the English language, whether it be speaking, writing, or reading. In the context of our review, we refer to LEP in spoken communication. It is important to note that LEP is not indicative of a person’s intelligence or capabilities, but rather reflects their current proficiency in the English language.
Spanish-speaking Populations	Those whose chosen language of communication is Spanish. This includes native, monolingual and multilingual speakers, multiple ethnic groups and a wide range of cultures.
Ad hoc interpreters	Individuals who provide interpretation services on an informal or spontaneous basis, without formal training or qualifications as professional interpreters. These individuals may include family members, friends, bilingual staff members, or volunteers who happen to be available at the time of need.
Language concordance	When a healthcare provider and a patient share the same primary language or effectively communicate with each other in a common language.
Health disparities	Preventable differences existing among socially disadvantaged populations in the burden of disease, injury, or healthcare access.
Health literacy	The ability to comprehend and analyze information and instructions related to medical care. This may include interpreting written text, symbols, charts, or diagrams. Limited health literacy can affect people of all ages, races, incomes, and education statuses; however, it disproportionately impacts lower socioeconomic groups.
Hispanic	Heritage, nationality, lineage, or country of birth or the birth of one’s parents or ancestors originating from Spain. Those who identify as “Hispanic” may be of any race.
Latino	Heritage, nationality, lineage, or country of birth or the birth of one’s parents or ancestors originating from Latin America, such as Cuba, Mexico, Puerto Rico, South America, or Central America.
Fluency	The flow of an individual’s speech, stemming from the Latin word “fluere,” meaning “to flow.” The speech follows a rhythm and does not contain inappropriate stops within sentences spoken.
Proficiency	An individual’s ability and comfort in using a language spontaneously and without rehearsal at a given time.
Spanish fluency	Fluency in the Spanish language.

## Review

Methods

Relevant studies were identified through a comprehensive search of electronic databases including PubMed, SageJournals, Science Direct, and Springer Link using the following keywords: “language concordance,” “health outcomes,” “Spanish,” “language barriers,” “primary care,” and “rural settings.” The search terms were combined using Boolean operators: "Spanish OR Hispanic" for ethnic identification, "language concordance AND health outcomes" to explore the relationship between language alignment and patient results, and "Spanish AND primary care AND language barriers" to narrow the focus to specific healthcare settings. Additionally, the reference lists of relevant publications and review articles were manually screened to identify further applicable studies.

Two independent researchers conducted the literature search between April 10, 2024 and May 31, 2024. Studies were included if they were published in English, peer-reviewed, focused on the relationship between language concordance and healthcare outcomes, and published within the last 20 years (2004-2024). Studies were excluded if they were case reports, not peer-reviewed, published in a language other than English, or published before 2004.

A total of 35 articles were initially identified. After applying the inclusion and exclusion criteria, 18 articles were included in the final review. Data extraction was conducted using a standardized form to capture key details, including study design, participant demographics, and major findings. 

Literature review

Achieving a comprehensive understanding of the influence of language concordance on healthcare outcomes requires a thorough examination of the multitude of factors that contribute to its complexity or hinder effective communication. The literature reviewed highlights the critical role of language concordance in patient care, emphasizing its impact on communication quality, shared decision-making, healthcare utilization and adherence, and patient satisfaction and trust. Additionally, the importance of language support systems in healthcare was examined, with a focus on the quality and use of interpreter services, the effectiveness of language access policies, and the assessment of clinician language proficiency. These recurring themes underscore the essential connection between language concordance and healthcare outcomes.

While Kansas serves as a focal point in this review, the insights gained are applicable to other rural areas in the United States where similar demographic changes and healthcare challenges are present. The lessons learned from Kansas can guide the development of targeted interventions and policies that address language barriers in rural healthcare settings more broadly.

Language Concordance in Patient Care

Impact on communication quality and shared decision-making: Effective communication is a cornerstone of quality healthcare delivery, encompassing themes of trust, mutual respect, compassion, and cultural competency and responsiveness, and it can be achieved through language concordance. Language concordance facilitated more patient-centered interactions, characterized by increased psychosocial talk and greater patient participation [3]. Direct communication in Spanish was associated with higher ratings of perceived opportunity to disclose concerns (4.91 v 4.62 on a 5-point scale; p = 0.001) which indicated that language concordance facilitated more open and effective communication between physicians and patients [14].

Physicians with higher Spanish-speaking ability were perceived as more welcoming of non-medical talk (r = 0.25, p < 0.01), emphasizing the importance of language proficiency in building rapport and establishing trust with patients [3]. Patients expressed greater confidence in physicians with higher language proficiency (4.84 v 4.51; p = 0.001). This finding suggested that effective communication in the patient's preferred language can enhance perceptions of physician competence and expertise, though whether these results could be applied to languages other than Spanish was deemed inconclusive due to the limitations of the study sample [14].

Physicians with higher language proficiency were regarded as more connected to patients (r = 0.32, p < 0.01) and exhibited improved communication skills, leading to lower frustration levels with patients’ communication (r = -0.25, p < 0.01) and potentially fostering better-shared decision-making. However, a significant limitation is the correlational nature of the study, which means that third variables, such as past interactions and longer relationships between some patients and their physicians, could have influenced patient satisfaction with care. Other confounding variables to communication, such as race or ethnicity and nonverbal cues of facial expressions and body movements, were unable to be evaluated within the limitations of this study [3].

Enhanced healthcare utilization and adherence: Language concordance has been linked to improved healthcare utilization patterns and adherence to medical recommendations. Patients with language-concordant primary care physicians (PCPs) exhibited lower overall healthcare utilization compared to patients without a language-concordant PCP (12.22 v 13.07; p < 0.001), including 9.8% fewer specialist visits (8.73 v 9.69; p < 0.001), 19.8% fewer inpatient stays (0.14 v 0.17; p < 0.001), and 4.1% fewer emergency department visits (0.31 v 0.32; p = 0.09). The increased likelihood of these patients visiting their PCP (5.2% more PCP visits; 3.04 v 2.89, p < 0.001) for preventative and follow-up care was identified as a possible contributing factor. When communication barriers were mitigated, patients were more motivated to seek regular healthcare. The increased frequency of primary care visits underscored the positive influence of language-concordant care. The use of interpreters to achieve language-concordant care was not available for measurement and may have had a confounding effect on the results [5].

Improved patient-provider communication through language-concordant care enhanced patients’ understanding of treatment regimens and increased adherence to prescribed medications [15]. Additionally, language-concordant care was shown to have fostered better participation among diabetic patients in diabetes-related self-care activities (1.4 days v 0.7 days per week, p = 0.01), which potentially contributed to improved health outcomes. As mentioned, unmeasured factors of communication such as nonverbal cues could have impacted scores and health behaviors [27].

Improved patient satisfaction and trust: Language concordance was associated with higher levels of patient satisfaction and trust [2,12-14,27]. Among every racial or ethnic category, non-English-speaking patients consistently reported more adverse care experiences compared to their English-speaking counterparts (p < 0.01) [13]. Patients who primarily spoke Spanish, regardless of ethnicity or English proficiency, reported more positive perceptions of providers' interpersonal style when language concordance was achieved [27]. Language concordance was associated with higher interpersonal processes of care scores, particularly in the domains of elicitation of patient concerns and responsiveness. This indicated that language concordance contributed to more patient-centered care experiences [12]. Furthermore, patients with language-concordant physicians expressed greater comfort levels (94.5%) and satisfaction (100%, n = 18) compared to those reliant on interpreters respectively (46.7%; 14.3%, n = 7). However, the small sample size and difference in group size limited the generalizability of these results [2].

Patients reported higher satisfaction (97.6% v 91.8%; p < 0.001) and perceived quality of care (63% v 25% reported top ratings of 5/5; p = 0.001) when communication occurred in Spanish directly with physicians rather than through interpreters. This finding indicated a preference for language-concordant care. As is the theme throughout many of the included studies, these results were from a population with the preferred language of Spanish, and further research is needed to determine if these findings are applicable to other languages, or different ethnic and cultural groups [14].

Language Support in Healthcare

Importance of interpreter services and language access policies: While language concordance has shown to be ideal, interpreter services offer an alternative to bridging communication gaps when direct concordance cannot be achieved. The effectiveness of interpreter services was demonstrated through an analysis of various care aspects such as doctor communication, office staff communication, and overall satisfaction with care [4].

Patients who both required and always had an interpreter available reported significantly higher scores across all measures. The mean doctor communication score for these patients was 6.04 points higher than that of patients who did not require an interpreter (SE=1.47; p < 0.001). Similarly, office staff communication scores were 5.29 points higher (SE=1.83; p < 0.001), and overall satisfaction with care was 3.65 points higher (SE=1.19; p < 0.01) when language needs were adequately met [4].

In contrast, patients who required an interpreter but did not have one available reported the lowest scores. This highlighted the negative impact of unmet language needs. These patients' doctor communication scores were 4.28 points lower (SE=1.42; p < 0.001), office staff communication scores were 3.78 points lower (SE=1.77; p < 0.05), and overall satisfaction with care was 2.39 points lower (SE=1.15; p < 0.05) when compared to patients who did not require an interpreter [4]. This disparity further emphasized the critical role of interpreter services in maintaining effective communication and patient satisfaction.

Despite the positive outcomes associated with in-person interpreter services, patients expressed feelings of disempowerment and discomfort with remote interpreting methods, such as telephone interpretation or video remote interpretation. These methods often created a sense of social distance, as patients referred to remote interpreters as ‘la máquina’ (‘the machine’) rather than as people. This lack of personal interaction contributed to increased inequities in care [22].

The quality of interpretation services also plays a significant role in patient care. The use of professional interpreters in healthcare appointments resulted in significantly fewer translational errors with potential clinical consequences (12%), compared to ad hoc interpreters (22%) or no interpreters (20%). This underscored the importance of ensuring interpreter quality and training. Flores et al. found that a minimum of 100 hours of training for interpreters, rather than years of experience, was associated with the most significant reduction in errors of potential clinical consequence (2% v 12%) and overall errors (median 12 v 32.5) [6]. Professional interpreters thus not only reduced the likelihood of errors but also contributed to improved patient satisfaction and technical quality of care [6,14]. However, the applicability of these findings to LEP patients and families whose primary language was not Spanish remained to be fully established [6].

Furthermore, federal regulations have mandated the provision of language assistance services to individuals with LEP and established a framework for promoting linguistic and cultural competency in healthcare delivery [7,8]. Nonetheless, inadequate language access policies in states like Kansas, where competency requirements for healthcare interpreters were lacking due to reliance on third-party interpreting agencies, may have exacerbated healthcare disparities for Spanish-speaking populations. This highlighted an urgent need for standardized interpreter training and comprehensive language access plans to address these disparities effectively [8].

Assessment of clinician language proficiency: Accurate assessment of clinician language proficiency is pivotal for effective communication with non-English-speaking patients [9,10,12,15]. Clinicians who self-assessed their proficiency as either “Very Good” or "Excellent" demonstrated higher reporting accuracy of their skills (SD: 7.0 and 3.5 respectively) compared to those who rated their proficiency as “Good”, “Fair”, or “Poor” (SD: 12.6, 16.4, and 33.2 respectively) [9]. The disparity in self-reported language proficiency among healthcare providers highlighted the necessity for clear policies regarding language use in healthcare settings.

Concerns about language proficiency were further compounded by the frequent reliance of the healthcare system on ad hoc interpreters or spoken Spanish by clinicians with low proficiency levels, which was defined as conversational at best. Clinicians often relied on ad hoc interpreters or used their spoken Spanish when they interacted with their patients in the following situations: when they discussed discharge instructions (48.4% and 0%, respectively), consented to procedures (16.7% and 4.2%), updated patients on clinical condition (45.5% and 15.2%), obtained medical history (50% and 6.3%), and during morning pre-rounds (27.6% and 62.1%). It is important to note that the data was collected using self-reported language proficiency, which potentially differed from actual tested abilities. This affirmed the need for standardized language assessments and the implementation of clear policies regarding the use of language skills and language services. Not only are these interventions imperative in ensuring the competency of language services offered by healthcare organizations, but they are also mandated by federal regulations [10]. By recognizing the importance of language concordance and implementing appropriate policies, healthcare organizations can enhance communication with non-English-speaking patients, ultimately improving healthcare quality and patient outcomes.

Discussion

Challenges and Recommendations for Healthcare Organizations

Patients with LEP faced barriers such as time-restricted interactions, discrimination, and inadequate interpreting services, which underscored the need for healthcare systems to address structural issues and provide culturally and linguistically appropriate care [16,22]. Despite the recognized benefits of language concordance and interpreter services, significant challenges persisted, which included inadequate training and certification of interpreters, variations in language access policies, and structural barriers within healthcare systems [7,8,22]. There is a need for the implementation and regulation of standardized interpreter training programs at the federal level. Healthcare organizations must move towards consistent use of certified interpreters and provide training for health professionals on how to interact with and effectively use interpreters. Improvements in interpretation services and the development of comprehensive language access plans should be prioritized to promote equitable care for linguistically diverse patient populations.

General Limitations

Several limitations existed in this research. A lack of standardized procedures across studies for determining patient satisfaction caused difficulties when performing comparisons. This led to a largely subjective data set without objective comparison. Another limitation was the definition of “language concordance” as it was defined in these papers. While language concordance was defined broadly as a patient and physician speaking in the same language, some studies may not have considered other factors that impacted communication with their definition of “language concordance” [2,10,15]. For instance, while a physician and a patient may speak to one another in the same language, dialects, cultural influences, and levels of language proficiency can make communication difficult, potentially compromising the quality of care and patient satisfaction [2,13]. Additionally, the studies varied in how they measured language proficiency and concordance, with some relying on self-reported measures from clinicians, which may not accurately reflect true proficiency levels [9,10].

Moreover, another significant limitation is the small number of studies that met the inclusion criteria. The limited sample size restricts the generalizability of the findings, and it may be premature to draw definitive conclusions about the impact of language concordance on healthcare outcomes. As more studies are conducted in this area, it will be essential to revisit these trends to provide a more comprehensive understanding of the subject [11,16].

Furthermore, the demographics of these studies also limit their applicability to the entire USA. Many of these studies were conducted in urban areas, which typically have more diverse populations and greater access to language services compared to rural areas. As a result, the application of these findings to other parts of the country, particularly rural settings, is difficult to defend. Rural areas often have fewer healthcare resources and a different demographic composition, which could influence the effectiveness of language concordance in improving healthcare outcomes [8,17].

The concentration of studies in urban areas raises questions about the external validity of the findings and underscores the need for further research in more diverse settings, including rural areas where healthcare access and language barriers may present unique challenges [7,17]. By expanding research to include a broader range of settings and populations, future studies can help determine whether the benefits of language concordance observed in urban areas can be replicated in other contexts.

Addressing these limitations in future research is crucial for developing a more comprehensive understanding of the effects of language concordance on healthcare delivery and patient outcomes.

Future Directions

While the current literature provides valuable insights into the impact of language concordance on healthcare outcomes, several avenues for future research remain unexplored.

Patient perspectives on language concordance: First, it is crucial to delve deeper into patient perspectives to understand the full impact of language concordance on healthcare experiences and outcomes. Future studies could employ qualitative methods, such as interviews and focus groups, or quantitative patient surveys to gather insights into preferences, experiences, and barriers related to language concordance. Understanding patient views can guide the development of more patient-centered language policies and practices [2,7,11].

Standardized assessment of clinician language proficiency: Another critical area for future research is the development of standardized assessment methods for clinician language proficiency. The current variability in self-assessment practices highlights the need for reliable and nuanced evaluation tools that can be applied across diverse healthcare settings [9,10]. Research should explore the design and implementation of these tools to ensure that language proficiency is accurately measured and that clinicians are adequately prepared to communicate with non-English-speaking patients [9,10,12].

Policy development and implementation: Exploring the development and implementation of clear policies regarding language use in healthcare settings is also essential. These policies should include guidelines for the use of interpreters, comprehensive training programs for clinicians, and mechanisms to ensure compliance with federal regulations [7,10,18,19]. Interventional studies could investigate the effectiveness of enhancing language concordance through such policies, examining outcomes related to patient care, safety, and satisfaction [10,12,19].

Cultural responsiveness in healthcare: In addition to language, the impact of culture on medical care warrants further investigation. The strong tie between language and culture underscores the need to consider cultural responsiveness when providing healthcare, especially to Hispanic and Latino communities [12,16]. Future research should explore how cultural factors such as beliefs, religion, diet, traditions, and norms influence medical compliance, utilization of services, and overall physical and mental health. This research can inform the development of culturally responsive care models that address the unique needs of diverse populations [12,14,16,22].

Utilization and accessibility of language services: Lastly, the utilization and accessibility of language services across healthcare settings must be examined, particularly focusing on availability, affordability, and the effectiveness of interpretation and translation services. Research should explore the barriers that linguistically diverse populations face in accessing these services and develop strategies to improve both access and utilization. Additionally, studies focusing on the integration of technology-based language services, such as telemedicine interpretation platforms and language translation applications, could offer valuable insights into how technology can enhance language access and communication in healthcare delivery [4,6-8,19,24,27].

By addressing these research gaps, future studies can better inform clinical practice, policy development, and healthcare delivery, ultimately aiming to improve patient-centered care, health equity, and healthcare quality for linguistically diverse populations.

## Conclusions

This review highlights Kansas as a representative case study, offering valuable lessons for addressing language barriers in rural healthcare settings nationwide. The challenges faced in Kansas are indicative of broader issues that, if addressed, can lead to more equitable healthcare outcomes for non-English-speaking populations across the United States.

The literature underscores the critical role of language concordance, effective communication, and cultural responsiveness in improving healthcare outcomes. It is important to identify and define these disparities so they may be used to inform health policy and legislation. While language barriers remain a significant challenge, interventions aimed at promoting language concordance, enhancing interpreter services, and addressing structural barriers within healthcare systems have the potential to mitigate disparities and advance health equity for diverse patient populations.
